# Enteric Disease Outbreaks Associated with Animal Contact — Animal Contact Outbreak Surveillance System, United States, 2009–2021

**DOI:** 10.15585/mmwr.ss7403a1

**Published:** 2025-05-22

**Authors:** Taylor Eisenstein, Marisa Wong, Grace Vahey, Ariana Perez Toepfer, Brigette Gleason, Katharine Benedict

**Affiliations:** ^1^National Center for Emerging and Zoonotic Infectious Diseases, CDC, Atlanta, Georgia; ^2^Oak Ridge Institute for Science and Education, Oak Ridge, Tennessee

## Abstract

**Problem/Condition:**

An estimated 450,000 enteric illnesses, 5,000 hospitalizations, and 76 deaths associated with animal contact occur each year in the United States. Enteric illnesses are diseases that affect the stomach or intestines and cause symptoms, such as diarrhea, nausea, or vomiting, and are typically transmitted from animals to humans through the fecal–oral route. Humans might encounter animal feces or bodily fluids through contact with the animal itself, the animal’s environment, or the animal’s food or water. Although outbreak-associated illnesses account for a small subset of all enteric illnesses linked to animal contact, data obtained from outbreak surveillance offer insights into the underlying epidemiologic factors contributing to illnesses, including the pathogens, animals, pathogen–animal category pairs, and settings of outbreaks associated with animal contact.

**Period Covered:**

2009–2021.

**Description of System:**

The Animal Contact Outbreak Surveillance System (ACOSS) was launched in 2009 in conjunction with the National Outbreak Reporting System (NORS), a web-based platform that includes reports of foodborne and waterborne outbreaks as well as enteric disease outbreaks transmitted by contact with environmental sources, infected persons or animals, or unknown modes. ACOSS encompasses animal contact outbreaks that are reported to CDC through NORS. Local, state, and territorial health departments voluntarily report animal contact outbreaks, which are defined as two or more enteric illnesses associated with a common animal source. Outbreaks can involve single or multiple states; CDC staff typically report multistate outbreaks on behalf of state and territorial health departments. ACOSS defines an animal source as an animal (including domestic and wild animals); an animal’s feces or bodily fluids (except milk and other fluids consumed as food, which are defined as foodborne sources); an animal’s fur, hair, feathers, scales, or skin; an animal’s food; or an animal’s environment, which includes places in which it lives and roams.

**Results:**

During 2009–2021, a total of 557 animal contact outbreaks of enteric disease were reported in the United States through ACOSS, accounting for 14,377 illnesses, 2,656 hospitalizations, and 22 deaths. Exposures were reported in all 50 states, Washington, DC, and Puerto Rico. During the period there were 393 single-state outbreaks and 164 multistate outbreaks. Although multistate outbreaks comprised 29% of all outbreaks reported through ACOSS, they accounted for 80% of illnesses, 88% of hospitalizations, and 82% of deaths. Among 474 outbreaks with a confirmed single etiology, *Salmonella* was the most common cause of outbreaks (248 outbreaks [52%]); these outbreaks accounted for the most outbreak-associated illnesses (11,822 [85%]), hospitalizations (2,393 [91%]), and deaths (17 [77%]). *Cryptosporidium* (108 outbreaks [23%]) was the second leading cause of confirmed, single etiology outbreaks, followed by *Escherichia coli* (63 [13%]) and *Campylobacter* (52 [11%]). The most common exposure locations among outbreaks with a single location reported were private home (168 [40%]) and farm or dairy (89 [21%]). Among 467 outbreaks for which an animal source could be attributed to a single animal category, ruminants (171 [37%]) were the most implicated animal category (with 75% of ruminant outbreaks attributed to cattle), followed by poultry (155 [33%]) and turtles (39 [12%]). Poultry were associated with the most outbreak-associated illnesses (9,095 [66% of illnesses resulting from outbreaks attributed to a single animal category]), hospitalizations (1,804 [70%]), and deaths (15 [83%]). Most outbreaks (130 [84% of all poultry outbreaks]) attributed to poultry had private home reported as at least one of the exposure locations (i.e., backyard poultry) and were responsible for nearly all poultry-associated illnesses (8,897 [98%]). The most common confirmed pathogen–animal pair was *Salmonella* and poultry (132 outbreaks), followed by *Cryptosporidium* and ruminants (88), and *Salmonella* and turtles (37). *Salmonella* and poultry accounted for the highest number of outbreak-associated illnesses (8,965), hospitalizations (1,790), and deaths (15).

**Interpretation:**

Animal contact outbreaks of enteric disease reported through ACOSS provide insights into the animals and etiologies causing outbreak-associated enteric illnesses as well as other outbreak characteristics, such as settings in which outbreaks occur. These findings can guide public health actions, developed in collaboration with specific populations (e.g., backyard poultry owners) and including interventions tailored to settings, such as private homes and farms or dairies, that are more commonly associated with animal contact outbreaks. The high proportion of outbreaks occurring in private homes identifies a potential gap in proper hygiene and enteric disease prevention knowledge among animal owners, including owners of backyard poultry, which might be considered by owners to be pets rather than livestock. Settings and populations linked to ruminants, poultry, and turtles (particularly cattle, backyard poultry, and small turtles, respectively) are important targets for public health interventions because of the high number of outbreaks and outbreak-associated illnesses associated with these animal sources. Furthermore, the disproportionate impact of multistate outbreaks reiterates the importance of a collaborative national response but also might reflect limited resources to investigate or report animal contact outbreaks at state and local levels.

**Public Health Action:**

Public health partners should continue to report animal contact outbreaks through ACOSS to inform evidence-based interventions tailored to specific animals, pathogens, populations, and settings. Strengthening the capacity of local, state, and territorial health departments to investigate and report animal contact outbreaks is critical to improving surveillance of animal contact outbreaks. Close collaboration between state, local, and Federal public health and agricultural partners nationwide is also key in investigating and responding to multistate outbreaks. An integrated One Health approach that leverages the expertise of animal, environmental, and public health partners can facilitate successful public health interventions aimed at preventing animal contact outbreaks.

## Introduction

Humans frequently encounter animals and are often encouraged to interact with them, whether at home or in venues designated for animal contact (e.g., petting zoos, farms, and pet stores). In the United States, an estimated 57%–70% of households own at least one pet (*1*). Although human–animal contact is mutually beneficial to persons and animals, including positive impacts on mental, social, and physical health, it can also facilitate spread of enteric disease (*2*). For example, *Salmonella* linked to backyard poultry and reptiles, such as bearded dragons and small turtles, has regularly caused multistate outbreaks (*3*–*5*).

In the United States, 450,000 human enteric illnesses, 5,000 hospitalizations, and 76 deaths associated with animal contact were estimated in 2012 to occur annually; of these illnesses, 14% are estimated to be associated with outbreaks (*6*). Enteric diseases can be transmitted between animals and humans (i.e., enteric zoonoses) via the fecal–oral route; humans might encounter animal feces or bodily fluids through contact with the animal itself, its environment, or its food or water.

This report summarizes single-state and multistate animal contact outbreaks of human enteric illness during 2009–2021, including animal categories, outbreak settings, and pathogen–animal pairs. Understanding epidemiologic characteristics of animal contact outbreaks can provide further insight into human illnesses caused by pathogens transmitted through animal contact and might enhance the ability of Federal, state, territorial, local, and tribal agencies, in collaboration with industry partners, to improve measures for reducing such illnesses. The findings from this analysis highlight the connection between animals, humans, and the environment by describing outbreaks of enteric illnesses in humans transmitted by direct or indirect contact with animals.

## Methods

### Data Source

Local, state, and territorial health departments voluntarily report enteric disease outbreaks associated with animal contact through the Animal Contact Outbreak Surveillance System (ACOSS) (https://www.cdc.gov/nors/about/acoss.html). ACOSS was developed in 2009 by CDC, with reporting incorporated into the National Outbreak Reporting System (NORS), a web-based platform that receives reports of foodborne and waterborne outbreaks as well as enteric disease outbreaks transmitted by contact with infected persons, animals, environmental sources, or unknown modes. Agencies use a standard form to report various data about the outbreak and the outbreak vehicle (e.g., date of first illness onset, number of illnesses, and exposure setting). This report includes all finalized reports of enteric disease outbreaks linked to animal contact in ACOSS as of January 18, 2023, in which the date of first illness onset occurred during January 1, 2009–December 31, 2021.

### Animal Contact Outbreak Definitions and Specifications

ACOSS defines an animal contact outbreak as two or more enteric illnesses linked to contact with a common animal source (*7*). An animal source is specifically defined as an animal; an animal’s feces or bodily fluids (except milk and other fluids consumed as food, as those are defined as foodborne sources); an animal’s fur, hair, feathers, scales, or skin; an animal’s food; or an animal’s environment (including places it lives and roams, such as coops, cages, aquariums, farms, schools, petting zoos, and pet stores). When exposure to an animal source occurs in a single state, the outbreak is classified as a single-state outbreak and is reported by staff members in that state; when exposure occurs in two or more states, the outbreak is classified as a multistate outbreak. CDC staff members typically report multistate outbreaks on behalf of state and territorial health departments.

Etiologies reported through ACOSS include bacterial, parasitic, and viral pathogens. Outbreak etiologies are further classified as unknown, suspected, or confirmed. Reporting agencies designate the outbreak etiology as confirmed or suspected based on available laboratory data. An outbreak is categorized as a multiple etiology outbreak if more than one genus of pathogen is reported.

Animals are identified as outbreak sources using one or more of the following types of evidence: epidemiologic, laboratory, traceback, environmental assessment, or other additional supportive information collected by investigators. Certain outbreak investigations do not identify a source, and, in these instances, the animal is reported as unknown. An outbreak is classified as animal contact with undetermined vehicle (i.e., undetermined animal source) when the type of evidence strongly suggests a common animal source, but a specific animal source is not identified (e.g., epidemiologic: event at a petting zoo). Animal vehicles were not differentiated as suspected versus confirmed sources in outbreak reporting until 2017; therefore, all implicated animals were included in this analysis, regardless of confirmed or suspected status.

The location where exposure to the animal source occurred is reported as the outbreak setting (e.g., a petting zoo, farm, or private home). For certain outbreaks, multiple settings might be reported. Backyard poultry outbreaks have been defined as outbreaks involving domesticated, noncommercial poultry; they are defined in this report as outbreaks involving poultry in which at least one location of exposure was indicated as the private home (*4*). Farms or dairies reported were assumed to be public venues for the purpose of this analysis.

Animals implicated in outbreaks were categorized based on a hierarchical scheme (*8*). ACOSS categorizes animals into three increasingly specific levels: biologic class, major group, and subgroup. For example, a wild dog would be classified as mammal (biologic class), canine (major group), and wild canine (subgroup). An animal category was assigned if a single animal was reported as the source or if all implicated animals in an outbreak belonged to the same category (e.g., an outbreak with only goats or both goats and sheep reported as the source would both be assigned the animal category ruminants). When an outbreak could not be assigned to a single, specific animal category, the outbreak was classified as “not attributed to a single animal category” (e.g., an outbreak with dogs and poultry reported as animal sources cannot be assigned to a specific animal category). For this analysis, the major group served as the primary analytic category.

For outbreaks reported through ACOSS as linked to pet food and treats, the animal that is the intended recipient of the food is considered the implicated animal source. For example, in an outbreak that was attributed to pig ear pet treats consumed by dogs, the implicated animal vehicle was reported as “dog” and “pet treats or chews” was selected under the appropriate pet food and treats question. Because the pet food or treats question was not added to the reporting form until 2017, many of these classifications relied on comments review.

### Data Analysis

Descriptive analyses were conducted using SAS (version 9.4; SAS Institute) and Microsoft Excel for Microsoft 365 Office (version 2022; Microsoft Corporation). Population-based reporting rates were calculated using 2009–2021 midyear population estimates for each state obtained from the U.S. Census Bureau (*9*). A manual review of the comments contained in outbreak reports was also performed by CDC staff members. Variables such as settings, animal sources, and pet food and treat types were reclassified when the comments contained relevant information. This activity was reviewed by CDC, deemed not research, and was conducted consistent with applicable Federal law and CDC policy.*

## Results

During 2009–2021, ACOSS received reports of 557 outbreaks, resulting in 14,377 illnesses, 2,656 hospitalizations, and 22 deaths (Figure) (Supplementary Table, https://stacks.cdc.gov/view/cdc/177769#tabs-3). The median number of outbreaks reported per year was 45 (range = 21–62 outbreaks). Single-state outbreaks were reported by 44 states and accounted for 393 (71%) outbreaks and 2,935 (20%) illnesses (median = four illnesses per outbreak; range = 2–205 illnesses). Of 2,935 ill persons in single-state outbreaks, 323 (11%) were reported as being hospitalized. Exposures for multistate outbreaks occurred in all 50 states, Washington, DC, and Puerto Rico. Multistate outbreaks accounted for 164 (29%) outbreaks and 11,442 (80%) illnesses (median = 40.5 illnesses per outbreak; range = 3–848 illnesses). Of 11,442 ill persons, 2,333 (20%) were reported as being hospitalized. The overall hospitalization rate (including multistate and single-state outbreaks) was 18%, ranging from 7% in 2010 to 22% in 2015.

**FIGURE:**
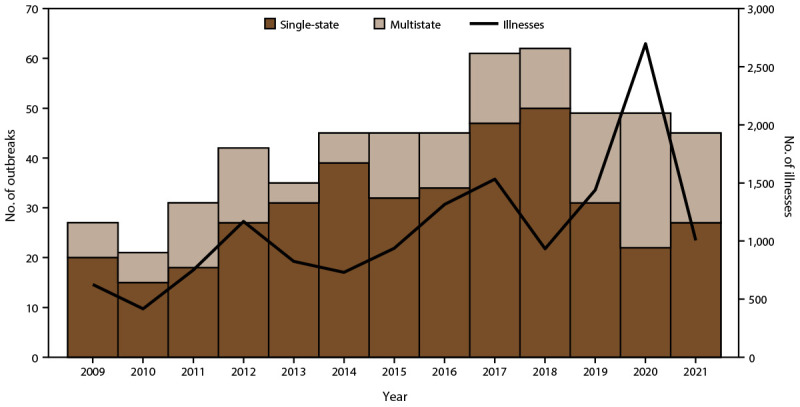
Number of enteric disease outbreaks and outbreak-associated illnesses associated with animal contact, by year* — Animal Contact Outbreak Surveillance System, United States, 2009–2021

### Etiologic Agents

A single confirmed etiology was reported for 474 outbreaks (85% of 557 total outbreaks), resulting in 13,941 illnesses, 2,623 hospitalizations, and 22 deaths (Table 1). Among those 474 outbreaks, *Salmonella* was the most reported pathogen (248 outbreaks [52%]) and was responsible for most reported outbreak-associated illnesses (11,822 [85%]), hospitalizations (2,393 [91%]), and deaths (17 [77%]). *Cryptosporidium* was the second most common confirmed single etiology reported and caused the second highest number of outbreak-associated illnesses (108 outbreaks [23%] and 913 illnesses [7%]), followed by Shiga toxin–producing *Escherichia coli* (STEC) (63 outbreaks [13%] and 790 illnesses [6%]) and *Campylobacter* (52 outbreaks [11%] and 410 illnesses [3%]). Although *Salmonella* and *Cryptosporidium* caused the most outbreak-associated illnesses in outbreaks with a confirmed single etiology (12,735 [91%]), *Salmonella* and STEC altogether resulted in the most hospitalizations (2,531 [96%]) and all 22 deaths; *Salmonella* outbreaks had the highest hospitalization rate (20% [2,393 of 11,822]). Altogether, bacteria were the most reported confirmed or suspected single etiology category (405 outbreaks [75%]), resulting in 13,160 illnesses (93%), 2,593 hospitalizations (98%), and all 22 deaths; all other outbreaks were attributed to parasitic pathogens.

**TABLE 1:** Numbers and percentages* of enteric disease outbreaks associated with animal contact, outbreak-associated illnesses, hospitalizations, and deaths, by etiology (confirmed or suspected) — Animal Contact Outbreak Surveillance System, United States, 2009–2021

Etiology^†^	Outbreaks				Illnesses				Hospitalizations				Deaths			
	CE	SE	Total	%*	CE	SE	Total	%*	CE	SE	Total	%*	CE	SE	Total	%*
**Bacterial**	**364**	**41**	**405**	**75**	**13,024**	**136**	**13,160**	**93**	**2,583**	**10**	**2,593**	**98**	**22**	**0**	**22**	**100**
*Salmonella*^§^	248	12	**260**	48	11822	36	**11,858**	83	2393	5	**2,398**	91	17	0	**17**	77
STEC^¶^	63	3	**66**	12	790	11	**801**	6	138	1	**139**	5	5	0	**5**	23
Campylobacter**	52	26	**78**	14	410	89	**499**	4	50	4	**54**	2	0	0	**0**	0
*Streptococcus*	1	0	**1**	0	2	0	**2**	0	2	0	**2**	0	0	0	**0**	0
**Parasitic**	**110**	**26**	**136**	**25**	**917**	**127**	**1,044**	**7**	**40**	**12**	**52**	**2**	**0**	**0**	**0**	**0**
*Cryptosporidium*^††^	108	26	**134**	25	913	127	**1040**	7	40	12	**52**	2	0	0	**0**	0
*Giardia*	2	0	**2**	0	4	0	**4**	0	0	0	**0**	0	0	0	**0**	0
**Single etiology total**	**474**	**67**	**541**	**97**	**13,941**	**263**	**14,204**	**99**	**2,623**	**22**	**2,645**	**100**	**22**	**0**	**22**	**100**
**Multiple etiologies^§§^**	**10**	**6**	**16**	**3**	**148**	**25**	**173**	**1**	**8**	**3**	**11**	**0**	**0**	**0**	**0**	**0**
**Total**	**484**	**73**	**557**	**100**	**14,089**	**288**	**14,377**	**100**	**2,631**	**25**	**2,656**	**100**	**22**	**0**	**22**	**100**

**Abbreviations:** CE = confirmed etiology; SE = suspected etiology; STEC = Shiga toxin–producing *Escherichia coli*.

* The denominator for the etiology percentages is the single etiology total. The denominator for single etiology and multiple etiologies is the total. Because of rounding, numbers might not add up to 100%.

^†^ Agents that are not known to cause enteric illness associated with animal contact are sometimes reported as confirmed or suspected etiologies.

^§^ *Salmonella* serotypes causing five or more outbreaks were Typhimurium (47 outbreaks), Enteritidis (27 outbreaks), I 4,[5],12:i:- (19 outbreaks), Infantis (14 outbreaks ), Braenderup (13 outbreaks), Montevideo (12 outbreaks), Hadar (10 outbreaks), Muenchen (7 outbreaks), Poona (six outbreaks), Sandiego (six outbreaks), Paratyphi B (five outbreaks), Indiana (five outbreaks), Pomona (five outbreaks), Mbandaka (five outbreaks), multiple serotypes (17 outbreaks), and unknown/other serotypes (eight outbreaks).

^¶^ STEC serogroups: O157 (44 outbreaks), O26 (four outbreaks), O111 (four outbreaks), O45 (one outbreak), O145 (one outbreak), multiple serogroups (nine outbreaks), and unknown serogroup (three outbreaks).

** *Campylobacter jejuni* (43 outbreaks), *Campylobacter coli* (five outbreaks), *Campylobacter* other species (one outbreak), *Campylobacter* multiple species (two outbreaks), and *Campylobacter* unknown species (27 outbreaks).

^††^ *Cryptosporidium parvum* (40 outbreaks) and *Cryptosporidium* unknown species (94 outbreaks).

^§§^ If at least two etiologies are confirmed in an outbreak, it is considered a confirmed multiple etiology outbreak; otherwise, it is considered a suspected multiple etiology outbreak.

### Location of Exposure

Among 545 enteric disease outbreaks associated with animal contact that had at least one reported location of exposure, a single location of exposure was reported in 417 (77%) outbreaks, accounting for 45% of outbreak-associated illnesses. Among outbreaks with a single location of exposure, 168 (40%) were linked to private homes, 89 (21%) to a farm or dairy, 36 (9%) to a festival or fair, and 28 (7%) to a petting zoo (Table 2).

**TABLE 2:** Numbers and percentages* of enteric disease outbreaks associated with animal contact and outbreak-associated illnesses, by location of exposure — Animal Contact Outbreak Surveillance System, United States, 2009–2021

Location^†^	Outbreaks No. (%)*	Illnesses No. (%)*
**Public venue**	160 (38)	1,513 (24)
Farm or dairy	89 (21)	580 (9)
Festival or fair	36 (9)	557 (9)
Petting zoo	28 (7)	340 (5)
Zoo or animal exhibit	2 (—)	8 (—)
Live animal market	2 (—)	16 (—)
Pumpkin patch	2 (—)	9 (—)
Museum	1 (—)	3 (—)
**Animal care setting**	25 (6)	186 (3)
Animal shelter or sanctuary	15 (4)	141 (2)
Veterinary clinic or animal hospital	8 (2)	25 (—)
Wildlife rehabilitation center	2 (—)	20 (—)
**Institutional setting**	28 (7)	280 (4)
School, college, or university	13 (3)	103 (2)
Camp	8 (2)	124 (2)
Child day care	5 (1)	26 (—)
Correctional facility	2 (—)	27 (—)
**Retail location**	10 (2)	241 (4)
Agricultural feed store	5 (1)	30 (—)
Pet store or other retail location	5 (1)	211 (3)
**Laboratory**	4 (1)	52 (1)
**Private home**	168 (40)	3,869 (61)
**Other setting^§^**	22 (5)	213 (3)
**Single location total**	417 (75)	6,354 (44)
**Multiple locations^¶^**	128 (23)	7,861 (55)
**No location reported**	12 (2)	162 (1)
**Total**	**557 (100)**	**14,377 (100)**

* The denominator for the location percentages is the single location total. The denominator for single location, multiple locations, and no location reported is the total. Because of rounding, numbers might not add to 100%. Dashes in parentheses signify <1%.

^†^ Several setting locations were grouped under subcategories based on author interpretation of the limited outbreak information reported through the National Outbreak Reporting System (i.e., Animal Contact Outbreak Surveillance System does not systematically collect data on the indicated categories [e.g., public venue]).

^§^ Settings classified as “Other” include livestock show or animal exhibition, poultry or turkey facility or processing plant, slaughter or meat packing plant, hatchery, long-term care facility, cattle truck rollover, and troubled youth ranch.

^¶^ Among outbreaks with multiple locations of exposure reported, private home was most commonly reported (110 outbreaks) as one of the locations of exposure. Agricultural feed store and private home were the settings most frequently reported together (68 outbreaks).

Among 545 outbreaks with at least one reported location of exposure, multiple locations of exposure were reported for 128 (23%) outbreaks, accounting for 55% of outbreak-associated illnesses. Among outbreaks with multiple locations reported, the most common exposure locations reported simultaneously were agricultural feed store and private home (68 outbreaks [53%]). Other locations reported in outbreaks with multiple settings included farm or dairy, pet store or other retail location, animal shelter or sanctuary, camp, and petting zoo (Table 2).

### Animal Sources

An animal source was reported in 505 outbreaks (91% of all outbreaks). Among 467 outbreaks (84% of all outbreaks) in which the implicated animal source could be attributed to a single animal category, ruminants (171 [37%]) were the most frequently implicated animal category, followed by poultry (155 [33%]) and turtles (39 [8%]) (Table 3). Among 171 outbreaks associated with ruminants, cattle were the most frequently reported animal (128 outbreaks [75%]) and accounted for 1,136 (70%) of 1,613 illnesses. Among 39 outbreaks associated with turtles, small turtles (shell length <4 inches) were most frequently reported (24 [62%]) and accounted for most illnesses (1,143 [86% of 1,323 illnesses]). The animal categories responsible for the most outbreak-associated illnesses were poultry (9,095 illnesses [66% of 13,806 illnesses resulting from outbreaks attributed to a single animal category]), ruminants (1,613 [12%]), and turtles (1,323 [10%]). Among outbreaks attributed to a single animal category, most hospitalizations (1,804 [70% of 2,563]) and deaths (15 [68% of 18]) reported through ACOSS were attributed to poultry.

**TABLE 3:** Numbers and percentages of enteric disease outbreaks associated with animal contact and outbreak-associated illnesses, by animal category — Animal Contact Outbreak Surveillance System, United States, 2009–2021

Animal category*	Outbreaks	Illnesses
	No. (%)	No. (%)
**Mammal**	233 (50)	2,475 (18)
Small mammals	15 (3)	235 (2)
Rodent^†^	5 (1)	54 (—)
Other small mammal^§^	10 (2)	181 (1)
Feline (cat)	4 (1)	9 (0)
Canine (dog)	31 (7)	481 (3)
Ruminants	171 (37)	1,613 (12)
Bovine (cattle)	128 (27)	1,136 (8)
Ovine (goat)	27 (6)	319 (2)
Caprine (sheep)	5 (1)	21 (—)
Multiple ruminants^¶^	11 (2)	137 (1)
Nonruminant hooved mammals	12 (3)	137 (1)
Swine (pig)	7 (1)	108 (1)
Equine (horse)	4 (1)	25 (—)
Multiple nonruminant hooved mammal**	1 (—)	4 (—)
**Bird**	158 (34)	9,130 (66)
Poultry	155 (33)	9,095 (66)
Baby chick, duckling, or both	42 (9)	1,078 (8)
Multiple poultry types	90 (19)	7,859 (57)
Other types of poultry^††^	23 (5)	158 (1)
Bird, not including poultry	3 (1)	35 (—)
**Reptile**	71 (15)	1,954 (14)
Squamates	29 (6)	546 (4)
Lizard	25 (5)	487 (4)
Snake	4 (1)	59 (—)
Testudines (turtle)	39 (8)	1,323 (10)
Turtle (shell length <4 inches)^§§^	24 (5)	1,143 (8)
Turtle (size unspecified)	15 (3)	180 (1)
Other reptile	3 (1)	85 (1)
**Amphibian**	2 (—)	241 (2)
Tailless amphibian (frog)	2 (—)	241 (2)
**Fish**	3 (1)	6 (—)
Pet fish	3 (1)	6 (—)
**Animal reported, attributed to a single animal category** ^¶¶^	467 (84)	13,806 (96)
**Animal reported, not attributed to a single animal category**	38 (7)	272 (2)
No animal reported***	52 (9)	299 (2)
**Total** ^¶¶^	**557 (100)**	**14,377 (100)**

* Animal categories are presented in the following order: biologic class (e.g., mammal), major group (e.g., small mammal), and subgroup (e.g., rodent). Dashes in parentheses signify <1%.

^†^ Guinea pig (five outbreaks).

^§^ Hedgehog (seven outbreaks), raccoon (two outbreaks), ferret (one outbreak).

^¶^ Goat and cattle (three outbreaks); cattle and sheep (three outbreaks); sheep and goat (two outbreaks); and sheep, goat, or cattle (one outbreak).

** Llama and pony (one outbreak).

^††^ Chicken (12 outbreaks), turkey (four outbreaks), baby chick or chicken (two outbreaks), other poultry (two outbreaks), duck (two outbreaks), pheasant (one outbreak).

^§§^ Small, juvenile turtles shed *Salmonella* more than adult turtles, particularly after experiencing stressors (e.g., handling and shipment); therefore, Federal and various state laws ban or limit the sale of small turtles (often defined as <4 inches in shell length) (**Sources:** Nichols M, Gollarza L, Palacios A, et al. *Salmonella* illness outbreaks linked to backyard poultry purchasing during the COVID-19 pandemic: United States, 2020. Epidemiol Infect 2021;149:e234 and CDC. Menu of state turtle-associated salmonellosis laws. Atlanta, GA: US Department of Health and Human Services, CDC; 2016).

^¶¶^ The denominator for the animal category percentages is the “animal reported, attributed to a single food category” total. The denominator for the “animal reported attributed to a single animal category,” “animal reported, not attributed to a single animal category," and “no animal reported” is the total. Because of rounding, numbers might not add up to the ”animal reported, attributed to a single animal category” total or the overall total.

*** The animal source was undetermined but epidemiologic, laboratory, traceback or environmental investigation, or other data strongly suggested animal contact as the mode of transmission.

Among 155 outbreaks attributed to poultry, 130 (84%) outbreaks had private home reported as at least one of the exposure locations (i.e., backyard poultry). Outbreaks linked to backyard poultry accounted for almost all human illnesses attributed to poultry (8,897 [98%]) and approximately half of all illnesses included in this report. Among 171 outbreaks attributed to ruminants (e.g., cattle, sheep, or goats), 89 (52%) outbreaks had farm or dairy reported as at least one of the locations of exposure. Among 39 outbreaks attributed to turtles, 35 (90%) outbreaks had private home reported as at least one of the exposure locations.

### Etiologic Agents and Animal Categories

The confirmed pathogen–animal pairs responsible for the most outbreaks with a single confirmed etiology were *Salmonella* from poultry (132 outbreaks), *Cryptosporidium* from ruminants (88 outbreaks), and *Salmonella* from turtles (37 outbreaks) (Table 4). *Salmonella* from poultry resulted in the most outbreak-associated illnesses (8,965), hospitalizations (1,790), and deaths (15) among outbreaks with a single confirmed etiology. *Salmonella* from turtles accounted for the second highest number of illnesses (1,318) among outbreaks with a single confirmed etiology, followed by *Cryptosporidium* from ruminants (769) and *Salmonella* from squamates (i.e., lizards and snakes) (536). *Salmonella* from turtles also accounted for the second highest number of hospitalizations (300). One death was attributed to each of the following confirmed pathogen–animal pairs: *Salmonella* and turtles, STEC and ruminants (goats and sheep), and *Salmonella* and small mammals (hedgehogs). The remaining deaths were attributed to confirmed, single etiology STEC outbreaks for which there was either no animal reported, or multiple animals reported that could not be attributed to a single animal category.

**TABLE 4:** Most common confirmed pathogen–animal category major group pairs resulting in outbreaks, outbreak-associated illnesses, hospitalizations, and deaths — Animal Contact Outbreak Surveillance System, United States, 2009–2021

Etiology	Animal major group*	Outbreaks (No.)	Illnesses (No.)	Hospitalizations (No.)	Deaths (No.)
**Top five pathogen–animal major group pairs resulting in outbreaks**					
*Salmonella*	Poultry	132	8,965	1,790	15
*Cryptosporidium*	Ruminants	88	769	29	0
*Salmonella*	Testudines	37	1,318	300	1
STEC	Ruminants	32	521	76	1
*Salmonella*	Squamates	25	536	118	0
**Top five pathogen–animal major group pairs resulting in outbreak-associated illnesses**					
*Salmonella*	Poultry	132	8,965	1,790	15
*Salmonella*	Testudines	37	1,318	300	1
*Cryptosporidium*	Ruminants	88	769	29	0
*Salmonella*	Squamates	25	536	118	0
STEC	Ruminants	32	521	76	1
**Top five pathogen–animal major group pairs resulting in outbreak-associated hospitalizations**					
*Salmonella*	Poultry	132	8,965	1,790	15
*Salmonella*	Testudines	37	1,318	300	1
*Salmonella*	Squamates	25	536	118	0
STEC	Ruminants	32	521	76	1
*Salmonella*	Tailless amphibians	2	241	45	0
**Pathogen–animal major group pairs resulting in outbreak-associated deaths**					
*Salmonella*	Poultry	132	8,965	1,790	15
*Salmonella*	Testudines	37	1,318	300	1
STEC	Ruminants	32	521	76	1
*Salmonella*	Small mammals	10	193	37	1

**Abbreviation:** STEC = Shiga toxin–producing *Escherichia coli.*

* Animals are categorized according to the animal categorization scheme (https://www.cdc.gov/nors/about/categorization.html).

### Pet Food and Treats

Pet food and treats were linked to 13 outbreaks (2% of all outbreaks), resulting in 459 (3%) illnesses, 72 (3%) hospitalizations, and one (5%) death. Among 13 outbreaks linked to pet food and treats, eight (62%) involved frozen or fresh feeder rodents or chicks, two (15%) involved pet treats or chews, two (15%) involved prepackaged pet food, and one (8%) involved homemade pet food. Outbreaks involving frozen or fresh feeder rodents or chicks caused the highest number of illnesses (213 [46%]), followed by pet treats or chews (197 [43%]), prepackaged pet food (34 [7%]) and homemade pet food (15 [3%]). Outbreaks linked to pet treats or chews were associated with dogs and caused the most hospitalizations (51 [71%]) among outbreaks linked to animal food. Of the two outbreaks involving prepackaged pet food, one was associated with dogs (31 illnesses [91% of all illnesses associated with prepackaged pet food]) and one was associated with hedgehogs (three illnesses [9%]). The single outbreak involving homemade pet food also was linked to live poultry; among other patients with exposure to backyard poultry, one patient reported making cat food at home using frozen rabbit (purchased online) and game hen.

### Multistate Outbreaks

During 2009–2021, there were 164 (29%) multistate outbreaks; however, these were responsible for 11,442 (80%) illnesses, 2,333 (88%) hospitalizations, and 18 (82%) deaths. Multistate outbreaks involved a median of 16.5 states, with a range of two to 49 states in which exposure occurred. The animal sources reported most frequently in multistate outbreaks were poultry (105 outbreaks [64%]) and turtles (28 [17%]). The animal sources responsible for the most outbreak-associated illnesses in multistate outbreaks were also poultry (8,803 illnesses [77%]) and turtles (1,262 [17%]).

## Discussion

This multiyear summary of data reported through ACOSS since its inception in 2009 demonstrates the value of public health surveillance of animal contact enteric disease outbreaks by identifying key pathogen–animal pairs, animal sources, and settings associated with outbreaks. The findings in this report underscore that *Salmonella* and poultry are substantial causes of enteric disease outbreaks associated with animal contact. Backyard poultry were associated with the highest number of outbreaks and illnesses among all animal contact outbreaks during the years reviewed in this report. Poultry have long been recognized as carriers of *Salmonella* species, and the number of backyard poultry–associated salmonellosis outbreaks has increased in recent years, along with the rising popularity of backyard flocks (*4**,**10*). Although the increase in cases might reflect an increase in outbreak detection because of enhanced molecular techniques (e.g., whole-genome sequencing), it nevertheless reiterates the persistent risk for *Salmonella* transmission between backyard flocks and their human families (*4*). As flock ownership grows in popularity, continued prevention efforts are necessary to communicate effectively with backyard poultry owners, particularly with families new to raising backyard poultry.

Turtles were the second most common animal source in multistate outbreaks and were responsible for the third highest number of outbreaks and illnesses in this report, despite Federal and various state laws that ban or limit the sale of small turtles (often defined as <4 inches in shell length) with limited exceptions (*11*). Small, juvenile turtles shed *Salmonella* more than adult turtles, particularly after experiencing stressors (e.g., handling and shipment) (*12*). Furthermore, children are more likely to handle small turtles because of their size and they often lack effective hand hygiene habits or knowledge, which increases the risk for *Salmonella* transmission from small turtles to humans (*13*,*14*). Although turtle size was not reported in all outbreaks reported through ACOSS, most turtle-associated outbreaks and illnesses were specifically associated with small turtles. The illegal sale of small turtles persists because of the difficulties of tracking and regulating vendors, specifically transient vendors and online retailers, which highlights the need for continued One Health^†^ efforts (*15*–*17*). States and localities also can take alternative preventative measures by establishing individual turtle-associated salmonellosis laws, potentially those mirroring various existing state regulations that limit turtle access around vulnerable populations (i.e., children) (*11*,*16*).

The findings in this report identify specific settings that are frequently reported in enteric disease outbreaks and highlight the importance of a One Health approach to disease mitigation and prevention. The most frequently reported location of exposure among all animal contact outbreaks during 2009–2021 was private home, which demonstrates a potential gap in enteric disease prevention knowledge among pet owners (including owners of backyard poultry and turtles) and further emphasizes the importance of improved sanitary practices when interacting with animals in private homes, regardless of whether animals are intended to be pets or livestock (*1*).

Public settings also have a crucial role in animal contact outbreaks; in this summary, farms and dairies, festivals or fairs, and petting zoos were the most frequently reported public venues among single-location outbreaks. Occupational exposure to animals among farm workers can cause enteric disease; as a result, preventive measures (e.g., appropriate personal protective equipment and handwashing technique) should be emphasized in the workplace (*18*). Recommendations from the National Association of State Public Health Veterinarians to mitigate the spread of illness from animals to humans (including both visitors and staff members) in public venues include improving facility design (e.g., making sure there is adequate separation between animal and nonanimal areas and appropriate handwashing equipment), educating persons on disease transmission risks and encouraging precautions (e.g., handwashing), and dissemination of public health messaging to a diverse audience (e.g., venue operators, associations and industry groups, and public health officials) (*19*).

An integrated One Health approach that leverages the expertise of partners in diverse sectors, including state and Federal governmental agencies, industry officials, public health experts, human and veterinary health care providers, and animal owners, can facilitate successful public health prevention measures for animal contact outbreaks in various settings (*19*). Cultivating partnerships that harness expertise across various groups improves awareness of existing materials and resources (e.g., compendia and materials from the National Association of State Public Health Veterinarians) while also refining message delivery methods to promote safer interactions between humans and animals.

Nearly one third of all outbreaks reported through ACOSS during 2009–2021 were multistate, accounting for approximately half of all outbreak-associated illnesses in this report. By contrast, multistate outbreaks have historically constituted a much smaller proportion of outbreaks and illnesses reported through NORS (*20*). Previous studies indicate that animal contact outbreaks are more likely to be multistate and of longer duration compared with foodborne outbreaks (*21*). This might result from a variety of factors, including a difference in intervention options. For example, contaminated food products have a regulatory structure through which products can be recalled and removed from commercial sale and further distribution whereas most purchased pets or animals do not have an analogous regulatory structure for recalls (*21*). More appropriate interventions for reducing illnesses linked to animal contact recognize that many enteric pathogens are normal components of healthy animals’ microbiomes (*21*). Close collaboration between Federal, state, territorial, and local public health and agricultural partners nationwide is essential to successfully investigate and respond to multistate outbreaks. The small number of single-state animal contact outbreaks relative to multistate overall compared with foodborne outbreaks might reflect limited resources for local, state, and territorial agencies to detect, investigate, or report these outbreaks. Nonetheless, strengthening capacity for public health officials in local, state, and territorial agencies to collect and report detailed outbreak information is critical to enhancing understanding of and preventing animal contact outbreaks through effective interventions.

## Limitations

The findings of this report are subject to at least five limitations. First, because ACOSS is a dynamic surveillance system, agencies can submit, update, or delete reports at any time; as a result, the results of this analysis might differ from previous or future reports. Second, the number of outbreaks is likely underestimated because reporting enteric disease outbreaks associated with animal contact is voluntary, and agencies might be limited in their ability to investigate or report outbreaks in their entirety and across jurisdictions. Certain outbreaks have an unknown animal source, and analyses and conclusions drawn from outbreaks with an identified animal source might not be representative of all animal contact outbreaks. Third, health departments might vary in how they define animal contact, particularly in how they solicit exposures to animal environments in which the ill person did not directly touch the animal (i.e., indirect transmission). Fourth, assumptions made in the absence of specific information might be subject to misclassification. Finally, a set of etiology confirmation guidelines specific to animal contact outbreaks does not exist as it does for foodborne outbreaks (https://www.cdc.gov/foodborne-outbreaks/php/investigating-outbreaks/confirming_diagnosis/index.html); as a result, reporting agencies might differ in their confirmation criteria.

## Future Directions

ACOSS continues to receive outbreak reports from state, local, Federal, and territorial partners. In 2023, NORS launched an updated form that includes new and modified fields for ACOSS. As part of the new form, ACOSS began systematically collecting functional purpose of animals implicated (e.g., pet or companion animal, backyard or residential livestock or poultry, and interactive exhibit animal). The new form also includes a section that asks what interventions (e.g., handwashing, animal quarantine, and vaccination) were implemented during the outbreak and at what point during the investigation (e.g., point of distribution of the suspected outbreak source, such as a shipping facility). In addition, insights into animal contact outbreaks might be gained by the enhanced efforts to collect information on occupational exposures, animal sources, and interventions, further informing future targets for outbreak prevention.

## Conclusion

This report summarizes key epidemiologic features of enteric disease outbreaks associated with animal contact during 2009–2021, including the etiologies, animals, and settings in which they occur. Public health agencies should continue to report enteric disease outbreaks associated with animal contact to inform interventions to prevent illnesses and enhance understanding of the interactions between humans, animals, and the environment. Strengthening the capacity of local, state, and territorial health departments to detect, investigate, and report these outbreaks is critical to improving surveillance. Outbreaks caused by contact with animals, especially those that occur across multiple states, demonstrate the need for a collaborative, multisectoral One Health approach that involves human, animal, and environmental health partners to ensure that persons can safely enjoy the benefits of interacting with animals.

## Footnotes

* 45 C.F.R. part 46.102(l)(2), 21 C.F.R. part 56; 42 U.S.C. Sect. 241(d); 5 U.S.C. Sect. 552a; 44 U.S.C. Sect. 3501 et seq.

† One Health is defined by CDC as “a collaborative, multisectoral, and transdisciplinary approach, working at the local, regional, national, and global levels, with the goal of achieving optimal health outcomes recognizing the interconnection between people, animals, plants, and their shared environment.” https://www.cdc.gov/one-health/about/index.html
